# Superseding the Hourglass Effect Toward the Successful Commercialization of Nanotechnology in the Medical Sciences – We Require a Change in Perspective

**DOI:** 10.7759/cureus.670

**Published:** 2016-07-05

**Authors:** Krishnan Chakravarthy, Frank Boehm, Wendy Sanhai-Madar

**Affiliations:** 1 Department of Anesthesia, Critical Care and Pain Medicine, Massachusetts General Hospital/Harvard Medical School; 2 The Johns Hopkins Institute for NanoBioTechnology, Johns Hopkins University; 3 Department of Chemistry, Lakehead University; 4 Department of Clinical Pharmacology, Duke University School of Medicine

**Keywords:** nanotechnology, nanomedicine

## Abstract

Nanotechnology and, specifically, nanomedicine has been touted as the next breakthrough technology for medical sciences. Although there are large advances being seen in the preclinical phases of development, there is still a paucity of viable and effective nanomedicine technologies in the clinical setting. We attempt to provide some suggestions as to the stumbling blocks of meaningful translation of this technology from the bench to the bedside. We give due consideration to the role of evidence-based medicine, regulatory pathways, and the commercialization efforts of nanomedicine at various stages in playing key roles in moving this technology into clinical use.

## Editorial

The nanoscale simply refers to any entity or feature that possesses dimensions of 10^-9 ^meters [1]. The potential, broad-based benefits of nanotechnology and nanomedicine to society are profound—alternative energy sources, safer food and water supplies, environmental science, safe and effective medicines, and consumer goods—and these are made possible by the inherent flexibility and unique characteristics possessed by nanomaterials. In medicine, multidisciplinary approaches have led to innovative products, such as targeted drug delivery systems, new molecular entities, nanorobotics operating in conjunction with artificial intelligence (AI), and point-of-care diagnostics. Similarly, nanotechnology and nanomedicine will require a multidisciplinary approach to realize their full potential and benefits to patients.

For over two decades, scientists and researchers have devoted significant resources to exploring the potential benefits of nanotechnology and nanomedicine. And while we may, at some point, envision a world where nanotechnology and nanomedicine impact our daily lives, there still remains a host of research and development (R&D) hurdles that must be overcome before this can be a reality. Bridging clinical gaps and translating nanotechnology from concept to clinical practice use will require a global effort, sustained funding, and multidisciplinary approaches.

Public-private partnerships represent a feasible mechanism whereby the resources and expertise of numerous stakeholders can be devoted to solving seemingly insurmountable challenges in bringing nanomedicine to patients in a timely and cost-effective manner [2]. Through collaboration, the benefits of nanomedicine can be seen in oncology, where precise delivery of chemotherapeutic drugs, directly to the lesion and in lower doses, may reduce toxic side effects and increase efficacy [3]. In light of the decreasing efficacy of currently available antibiotics, novel antivirals and antibacterial agents may be developed. Growth mediators, coupled with nanotechnology, may enhance and expedite tissue and cellular regeneration, emerging as a standard component of trauma and emergency department units. Smart nanotechnology platforms may have the capacity to carry out functions, ranging from simple intracellular detectors to more complex actions, such as seeking out tumor cells for eradication, and the repair of damaged organelles and/or entire cells [4].

As patients and physicians, we must take responsibility for educating ourselves about the benefits and potential risks that may accompany novel nanotherapies and nano-engineered medical devices. Ongoing investments of $1.5 billion in the National Nanotechnology Initiative (NNI) through the US government in the fiscal year 2016, and further expenditures through the private sector, will likely expand the global nanotechnology medical market to an estimated $177.6 billion by 2019. This translates to increasingly broad-based consumption by the general public, which underscores the need for educating the lay public about the potential applications and limitations of nanotechnology and nanomedicine as knowledge and understanding will decrease the fear associated with the adoption of novel, nanoengineered products.

Despite ongoing government and private investments, and a growing overall market, the discipline of nanomedicine continues to be plagued by challenges in R&D and consumer acceptance [2]. This is especially pertinent to drugs and drug-delivery systems that have long R&D timelines and require extensive investments. Furthermore, these therapies must meet current good manufacturing practices as well as clinical data supporting safety and efficacy criteria before they can be approved for the US market by the Unites States Food and Drug Administration (FDA). The significant financial investments, scientific and clinical hurdles, and a relative lack of transparency and specific guidelines from regulators, like FDA, have all contributed to stunted growth in the fields of nanotechnology and nanomedicine. An undesirable outcome of this stunted growth over the years has been a decrease in venture capital and other private investments in these fields as uncertainty over returns of investments looms. As such, it is not surprising that a significant portion of R&D into nanoengineered medical products occurs within laboratories of major universities and is siloed in start-up companies hoping for the transition of their embryonic technologies and compounds to larger biotech and pharmaceutical companies. Many larger pharmaceutical companies, on the other hand, have been employing a wait-and-see strategy to nanotechnology product development, even as they provide potential exit strategies to smaller companies with a desire to transition small molecules for subsequent clinical development. Other prospective nanoengineered medical products fall prey to the immense chasm, created by a combination of a risk-averse investment community, an unclear regulatory pathway, and a high level of consumer ignorance about the potential benefits of nanotechnology and nanomedicine.

Still, there is hope to sustain investments and support for nanotechnology and nanomedicine through improved public and private awareness. An investment community that is sufficiently educated in terms of how nanotechnology may be applied to specific medical conditions will undoubtedly better appreciate the pathways and timeline to commercialization and be cognizant of the value of investing in those early proof-of-concept projects that show promise with early, positive clinical outcomes. This will be of increasing importance in attempting to align the growing disparity between bench innovation versus commercial viability within the nanomedicine field. In this light, the FDA will play a critical role in helping to usher in new nanomedical products into the marketplace by providing clear guidance and evidence-based approaches to product development.

Some of the regulatory concerns of nanotechnology includes, but are not limited to, effectively bridging preclinical, clinical, and manufacturing phases of medical product development; quantification of distributed nanoparticles in the body following systematic administration (bio-distribution); solubility issues and aggregation in complex biological systems; and the potential for nanoparticles to negotiate biological barriers. Furthermore, quantification of the long-term effects of nanomaterials on patient health remains an elusive goal. Needless to say, there exist many unanswered, multivariate problems in nanotechnology and nanomedicine, and no one entity possesses all the know-how or resources to address these questions. The authors propose that bridging existing scientific and clinical gaps will require leveraging and collaboration among stakeholders: regulators, academicians, private industry, professional organizations, and government. Moreover, the development of nano-engineered medical products must take a patient-focused approach in which real-time feedback is provided by patients and clinicians in a way that can inform product development. Strategic collaboration across multiple disciplines, creative business models, and funding mechanisms are likely to provide the most feasible way forward to advancing nanotechnology and nanomedicine for patient benefit. 
